# Quantifying Compassion Fatigue in Ancillary and Clinical Staff in an Adult Emergency Department

**DOI:** 10.5811/westjem.2022.8.57733

**Published:** 2022-10-18

**Authors:** Melissa Bales, Katelyn DeAlmeida, Courtney E. Oei, David Hampton, Nicole L. Bohr

**Affiliations:** University of Chicago Medicine, Department of Emergency Medicine, Chicago, Illinois

## Abstract

**Introduction:**

Emergency department (ED) staff are at a high risk for compassion fatigue (CF) due to a work environment that combines high patient acuity, violence, and other workplace stressors. This multifaceted syndrome has wide-ranging impacts which, if left untreated, can lead to adverse mental health conditions including depression, anxiety, and substance use disorders. However, the majority of studies examining CF look solely at clinicians; as a result, there is little information on the impact of CF across other roles involved in supporting patient care. We conducted this study to establish the prevalence of CF across both clinical and non-clinical roles in the adult ED setting.

**Methods:**

For this single institution, cross-sectional study, all full- and part-time ED staff members who worked at least 50% of their shifts in the ED or within the adult trauma service line were eligible to participate. Using the Professional Quality of Life Scale, which measures CF via compassion satisfaction (CS), burnout (BO), and secondary traumatic stress (STS), we assessed for group differences between roles using non-parametric one-way ANOVA.

**Results:**

A total of 152 participants (response rate = 38.0%) completed the survey. This included attending physicians (n = 15, 9.7%), resident/fellow physicians (n = 23, 15.1%), staff nurses (n = 54, 35.5%), emergency technicians (n = 21, 13.8%), supportive clinical staff (n = 28, 18.4%), and supportive ancillary staff (n = 11, 7.2%). Across all roles, the majority of respondents had average levels of BO (median = 25.0, interquartile range [IQR] 20.0–29.0) and STS (median = 23.0, IQR 18.0–27.0) coupled with high levels of CS (median = 38.0, IQR 33.0–43.0). There was a difference in CS by role (P = .01), with nurses reporting lower CS than attending physicians. Secondary traumatic stress also differed by role (P = .01), with attending physicians reporting lower STS than both emergency technicians and nurses. Group differences were not seen in BO.

**Conclusions:**

Rates of compassion fatigue subcomponents were similar across all ED team members, including non-clinical staff. Programs to identify and mitigate CF should be implemented and extended to all roles within the ED.

## INTRODUCTION

Emergency department (ED) staff today face unique challenges that may position them to be at an increased risk for developing compassion fatigue (CF).^1^ The ED environment itself is stressful—high pressured and fast paced. Staff in the ED often encounter high patient acuity, excessive workloads, and crowding.^2^,^3^ In addition, violence/abuse directed at staff is commonly experienced in the ED. One study found that over 80% of ED staff reported violence/abuse from their patients and/or patients’ families.^3^ The ED staff are also the frontline workers who most consistently experience the failures of a broken healthcare system. Of the 130 million ED visits per year in the United States, only about 12% are admitted to the hospital.^4^ Many of these visits are patients who frequently seek emergency care for non-emergent concerns. These patient encounters often stem from lack of access to primary care. Caring for these patients has been associated with increased feelings of hopelessness and CF among ED staff.^5^,^6^

Since 2010, the Professional Quality of Life Scale (ProQOL) scale has been the predominant CF measurement tool. This validated instrument individually assesses compassion satisfaction (CS), burnout (BO), and secondary traumatic stress (STS) to capture CF.^7^ Compassion satisfaction is defined as the gratification one feels secondary to the quality of their work and the care they provide.^7^ Alternatively, BO describes the feelings of hopelessness and frustrations that one experiences over time due to the perceived inability to do their job to the best of their ability. It is influenced by heavy workloads and unsupportive environments.^7^ Finally, STS is the secondhand distress one experiences when their job requires helping those who have experienced exceptionally traumatic events.^7^,^8^ While CS helps combat CF, BO and STS contribute to its development.

Compassion fatigue in healthcare workers has been extensively studied over the past 20 years; this includes studies among ED nurses,^9^–^11^ emergency physicians,^10^ and social workers in EDs.^10^ However, no studies have looked beyond those providing clinical care to capture CF in those working in supportive roles within the ED (such as environmental service staff, public safety officers, and registration staff). It is vital to capture the impact of CF across ED service lines, particularly given the workplace challenges present for all staff. In fact, one study found no statistically significant differences in CS, BO, or STS despite varying levels of patient contact between clinical roles.^10^ Therefore, it stands to reason that all ED staff could also be at risk for CF. To address this critical gap, we sought to capture the prevalence of CF in all employees who worked in the ED and identify group differences in CF by role.

## METHODS

Following local institutional review board approval in January 2020, we distributed a survey to all eligible staff working in the adult ED at a tertiary academic care facility with a Level I trauma center via REDCap (Research electronic data capture) hosted at the University of Chicago.^12^ This single-institution, cross-sectional study was conducted in January 2020. All full- and part-time ED staff members who worked at least 50% of their shifts in the ED or within the adult trauma service line were eligible to participate. This included attending and resident physicians, nurses, emergency technicians, supportive clinical staff, and support ancillary staff.

**Population Health Research Capsule**

What do we already know about this issue?

*Emergency department (ED) staff are at high risk for compassion fatigue (CF); however, most studies have only examined CF rates among clinical staff*.

What was the research question?

*We sought to quantify CF in both clinical and ancillary ED staff and identify group differences by role*.

What was the major finding of the study?

*Rates of CF subcomponents were similar across all ED team members, including non-clinical staff*.

How does this improve population health?

*Recognizing that CF impacts all staff in the ED, including those in non-clinical roles, will help institutions better address the issue and thereby improve patient care*.

For the purposes of this study, supportive clinical staff included respiratory therapists, radiology technicians, chaplains, and social workers. Support ancillary staff included environmental services staff, public safety officers, and registration staff. Staff members were excluded from study participation if they were 1) temporary/agency staff; and/or 2) hired within three months of the study start date. Survey completion was regarded as participants’ informed consent. Staff members who completed the survey were invited to participate in a random drawing to win one of 30 $50 gift cards.

We captured CF via the ProQOL version 5 scale, a validated tool that has been used in multiple research studies to quantify the prevalence and degree of CF in various healthcare roles.^7^ Scores range from low, average and high in each subcategory (CS, BO and STS). If the sum of an individual’s scores is 22 or less, this indicates low levels of that particular subcategory; between 23–41 indicates average levels, and 42 or higher indicates high levels.^7^ Those with high CS and low to moderate BO and STS may indicate low levels of CF, while individuals with low levels of CS and high levels of BO and STS could indicate higher levels of distress.^7^ We also collected demographic information, including age, gender, job title, number of years worked in the ED, years of trauma experience, and proximity of their place of residence to the hospital.

Data cleaning and analysis was completed using SAS software version 9.4 (SAS Institute, Cary, NC). We removed outliers, defined as data points beyond three standard deviations of the mean, due to likelihood of erroneous data entry. Patterns of missing data were assessed to ensure randomness; scale means were imputed for each individual in scales with less than 25% missing data. We calculated demographic frequencies and ProQOL-5 subscale summary statistics for the entire sample and by role. Group differences in ProQOL-5 subscale scores between roles were examined using nonparametric one-way ANOVA. Statistical significance was defined as *P* ≤ .05 with confidence intervals were set at 95%.

## RESULTS

**Figure 1:**
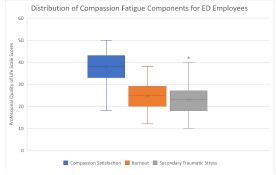
Boxplot demonstrating distribution of compassion fatigue components for emergency department employees.

Of the approximately 400 eligible staff members, 152 completed the survey, yielding a 38.0% response rate. Staff nurses (n = 54, 35.5%) and supportive clinical staff (n = 28, 18.4%) were the most common ED roles represented in this sample (Table 1). The majority were women (n = 94, 62%) and between the ages of 25 to 44 (n = 118, 78%). Most participants worked either 31–40 hours (n = 68, 45%) or greater than 40 hours (n = 74, 49%) per week; only 10 (7%) staff members worked 30 hours or less. Across all roles, half of respondents (n = 76) had average levels of BO (median = 25.0, IQR 20.0–29.0) and STS (median = 23.0, interquartile range [IQR] 18.0–27.0) (n=89, 58%) (Figure 1). The median CS score was 38.0 (IQR 33.0–43.0). There was a significant difference in CS by role (*P* = .02), with nurses reporting significantly less CS (median = 35.5) than attending physicians (median = 41.7) (Table 2). Secondary traumatic stress also differed by role (*P* =.02), with attending physicians reporting less STS (median = 18.0) than both emergency technicians (median = 25.3) and nurses (median = 23.4). Group differences were not seen in BO.

## DISCUSSION

In this study, which was conducted prior to the COVID-19 pandemic, average-to-high levels of CS and low-to-average levels of BO and STS were found across clinical and non-clinical roles within the ED. In the subgroup analysis, we demonstrated statistically significant differences. Attending physicians reported significantly higher levels of CS and lower levels of STS than nurses. This is inconsistent with findings from two other pre-pandemic studies comparing these roles using the ProQOL-5.^10^, ^13^ In both those studies, significant differences in subcategories between clinicians were not noted. However, of those studies one focused on palliative care staff,^13^ while the other measured CF subcomponents in strictly pediatric ED staff.^10^

Multiple factors may influence this inconsistency. A low level of managerial support, for example, has previously been associated with lower CF; this may account for the variation in reports across units, disciplines, hospitals, and studies.^14^ With that being said, population variations between studies have additionally made it difficult to draw general conclusions when making comparisons of CF between roles.^15^ This significance in CS and STS scores between nurses and attending physicians pre-pandemic adds to the growing body of literature analyzing the effects of working in the ED on each role.^10^, ^14^, ^16^, ^17^ The presence of CS, BO, and STS in supportive ancillary staff had previously never been examined. It is our assumption that this may be due to perceived lack of exposure to traumatic events or an under-appreciation of their impact on healthcare. However, there is potentially unanticipated indirect exposure, including the aftermath of seeing trauma patients, cardiac resuscitations, and patient death.

Given the overwhelming influx of patients during the COVD-19 pandemic, the increased exposure to death due to lack of treatment, direct exposure to the virus, and organizational issues such as lack of personal protective equipment, ED staff members have experienced increased levels of trauma overall.^18^–^20^ With the timing of our study, we were able to capture these pre-pandemic levels of CF and then extend the study to capture CF measures during the pandemic; those findings will be reported in a future publication.

It remains unclear how the pandemic has impacted supportive clinical staff and supportive ancillary staff. Some of these roles were reduced in the early days of the pandemic to decrease general population exposure; in addition, occupational resources for these roles were cut back due to financial constraints and to limit disease spread. With this reduction in resources to cope, supportive clinical and ancillary staff may have been at increased risk. Many institutions have offered education on self-care techniques and reinforced the availability of services such as employee assistance programs for clinical staff members. Given our results, institutions should be encouraged to extend similar resources to these supportive roles to help mitigate CF across all roles.

## LIMITATIONS

There are several limitations to our study. As previously stated, our response rate was 38%; however, the rate of participation for each role was representative of the overall distribution. Our survey was a self-selective process; therefore, because we were unable to capture those individuals who elected not to participate our scores may underrepresent the true prevalence of CS, BO, and STS. We hypothesize that those who did not participate may be more likely to be suffering from high levels of CF and, therefore, experiencing an indifference that prohibited study participation. Finally, this study was conducted in a single institution. The patient population and encounters experienced in our ED may not be identical to those seen at other locations. Our results may represent a bias toward a trauma center with a high penetrating injury rate and one without an established protocol to mediate staff CF.

## CONCLUSION

Compassion fatigue has the potential to be experienced by all trauma center service lines. Its presentation may be under-appreciated in service lines traditionally not associated with direct medical care. This lack of appreciation can result in a dysfunctional work environment, poor work performance, and career-limiting behaviors. There appears to be an internal element within institutions by the variations seen between studies. More research comparing roles across units may help clarify these differences. Additionally, the impacts of CF on supportive staff should continue to be investigated further to understand the impacts that COVID-19 had on these roles. Organizational recognition and support to create and implement protocols mitigating CF across all disciplines may lead to a greater understanding of its prevalence and opportunities for interventions.

## Figures and Tables

**Table 1:** Demographics and descriptive characteristics of emergency department staff.

	Resident/Fellow/Advanced Practice Nurse (n=23)		Attending Physicians (n=15)		Nurses (n = 54)		Emergency Room Tech/Medical Assistant (n=21)		Supportive Clinical Staff (n=28)		Supportive Ancillary Staff (n=11)		*P*
					
	n	%	n	%	n	%	n	%	n	%	n	%	
Gender													<.001
Male	6	26.0	13	86.9	15	27.8	12	57.1	8	28.6	4	36.4	
Female	17	73.9	2	13.3	39	72.2	9	42.9	20	71.4	7	63.6	
Age													<.001
<35	21	91.3	2	13.3	29	53.7	16	76.2	9	32.14	4	36.4	
35–54	2	8.7	7	46.7	23	42.6	4	19.1	18	64.3	7	63.6	
> 55	0	0.0	6	40.0	2	3.7	1	4.8	1	3.6	0	0.0	
Hours per week													<.001
<30	1	4.4	1	6.7	2	3.7	4	19.1	2	7.1	0	0.0	
30–50	4	17.4	1	6.7	48	88.9	16	76.2	25	89.3	10	90.9	
>50	18	78.3	13	86.7	4	7.4	1	4.8	1	3.6	1	9.1	
Years in role													.004
<2	14	60.9	4	26.7	13	24.1	11	52.4	10	35.7	2	18.2	
3–10	9	39.1	4	26.7	26	48.2	9	42.9	14	50.0	6	54.6	
>10	0	0.0	7	46.7	15	27.8	1	4.8	4	14.3	3	27.3	
Years in trauma													<.001
<2	15	65.2	0	0.0	25	48.1	11	55.0	17	60.7	7	63.6	
3–10	8	34.8	5	33.3	18	34.6	8	40.0	8	28.6	4	36.4	
>10	0	0.0	10	66.7	9	17.3	1	5.0	3	10.7	0	0.0	
Miles from job													.005
<5	9	39.1	1	6.7	7	13.2	5	23.8	3	10.7	3	27.3	
6–10	11	47.8	7	46.7	13	24.5	4	19.1	7	25.0	1	9.1	
>10	3	13.0	7	46.7	33	62.3	12	57.1	18	64.3	7	63.6	

**Table 2:** Compassion fatigue scores for emergency department staff via the Professional Quality of Life Scale. Version 5.

	Resident/Fellow/Advanced Practice Nurse (n=23)		Attending Physicians (n=15)		Nurse (n=54)		Emergency Room Tech/Medical Assistant (n=21)		Support Clinical Staff (n=28)		Support Ancillary Staff (n=11)		*P*
					
	Mean (STD)	Range	Mean (STD)	Range	Mean (STD)	Range	Mean (STD)	Range	Mean (STD)	Range	Mean (STD)	Range	
CS	39.6 (5.8)	29.0–49.0	41.7 (5.6)	30.0–50.0	35.5 (5.8)	22.0–50.0	38.1 (6.4)	25.0–50.0	38.1 (8.1)	18.0–49.0	39.0 (6.5)	29.0–49.0	.010
BO	24.0 (6.0)	15.0–35.0	21.5 (4.8)	14.0–31.0	25.3 (5.5)	12.0–34.0	26.5 (5.1)	18.0–35.0	24.8 (7.6)	12.0–38.0	24.2 (5.7)	13.0–31.0	.210
STS	22.6 (6.1)	10.0–33.0	18.0 (4.5)	12.0–27.0	23.4 (5.1)	11.0–33.0	25.3 (6.6)	13.0–38.0	23.1 (7.3)	14.0–42.0	21.6 (5.7)	13.0–30.0	.010

*CS*, compassion satisfaction; *BO*, burnout; *STS*, secondary traumatic stress.
